# Gluten Contamination of Rice: Analytical Testing *vs*. Consumer Perception – Is Rice Really Gluten Free?

**DOI:** 10.17113/ftb.63.03.25.9246

**Published:** 2025-09-24

**Authors:** Martina Bituh, Mihaela Gulin, Ksenija Marković, Ines Panjkota Krbavčić, Nada Vahčić

**Affiliations:** 1 University of Zagreb Faculty of Food Technology and Biotechnology, Pierottijeva 6, 10000 Zagreb, Croatia; 2 Graduate student at University of Zagreb Faculty of Food Technology and Biotechnology, Pierottijeva 6, 10000 Zagreb, Croatia

**Keywords:** gluten-free diet, rice, consumer perception, ELISA, food safety, Croatian market

## Abstract

**Research background:**

Gluten contamination is the main concern of those who follow a gluten-free diet. Although rice is naturally gluten-free, previous studies have identified gluten contamination of rice that can occur during processing, storage, handling or cooking. As a result, consumer confidence may be affected, emphasising the need to examine how these concerns shape their risk perceptions and influence their subsequent decisions. This study aims to evaluate: (*i*) the perceived risk of gluten contamination among gluten-free diet followers and (*ii*) the actual presence of gluten contamination in commercially available rice on the Croatian market.

**Experimental approach:**

This cross-sectional study combined survey methods and laboratory analysis. An online questionnaire was used to assess the perceived risk of gluten contamination in rice among individuals following a gluten-free diet (*N*=66). The presence of gluten in forty-one samples of white, brown and parboiled rice from six producers on the Croatian market was then analysed using an enzyme-linked immunosorbent assay (ELISA).

**Results and conclusions:**

Laboratory assays failed to detect gluten in any of the rice samples (limit of quantification 5 mg/kg), yet 54 (82 %) respondents expressed high uncertainty about the risk of gluten contamination in rice. These results show a significant discrepancy between consumer perception and scientific evidence. They emphasise the need for improved communication and clearer labelling to build consumer trust and support informed dietary choices.

**Novelty and scientific contribution:**

This study highlights the gap between the perceived and actual risk of gluten contamination in naturally gluten-free food and emphasises the importance of addressing consumer concerns through better education and transparent product information.

## INTRODUCTION

Rice is a staple food for millions of people around the world, and its high nutritional value and versatility make it a popular part of many diets (*1*). It is naturally free of gluten, a protein found predominantly in wheat, barley and rye, which should be avoided by individuals with coeliac disease to avoid damaging immune responses (*2*), as well as those with non-coeliac gluten sensitivity. Although coeliac disease affects approx. 1 % of the global population (*3*), the gluten-free market has grown far beyond this group (*4*). This growth is largely driven by individuals who choose a gluten-free diet for various other reasons, including perceived health benefits, weight management or adherence to popular dietary trends (*5*-*7*).

Rice should therefore be regarded as compatible with a gluten-free diet, yet rice, like other nominally ’gluten-free’ foods can become contaminated with gluten during processing, storage, handling and cooking (*8*). In fact, gluten has been found in commercial samples of not only rice, but also other grains naturally free of gluten, including corn, oats and buckwheat (*9*, *10*). Likewise, it has been detected in foods not naturally gluten-free but manufactured or processed to remove gluten and labelled as ’gluten-free’ (*11*–*15*). The unintended presence of gluten in products that are either naturally gluten-free or explicitly labelled as gluten-free raises significant concerns for consumers on a gluten-free diet. They increase the perceived risk of gluten exposure, which may influence their future food choices by leading to increased caution and mistrust of food labelling and safety guarantees. This highlights the continuing need for vigilance and systematic testing of gluten-free foods (*16*, *17*).

Reading and interpreting food labels is crucial for people on a gluten-free diet as they rely on this information to ensure the safety of their food choices. Food products whose normal gluten content has been reduced or eliminated through substitution of ingredients or through specific procedures of preparation or processing may bear the labels ’gluten-free’ (less than 20 mg/kg) or ’very low gluten’ (less than 100 mg/kg) in the European Union, as defined by EU Commission Implementing Regulation 828/2014 (*18*) based on Codex Alimentarius Standard (*19*). According to the Regulation, such claims must be presented in a way that does not mislead or confuse consumers. In addition to labelling, many gluten-free products, especially those intended for people with coeliac disease, carry voluntary certification marks such as the crossed grain symbol, which guarantees compliance with the AOECS (Association of European Coeliac Societies) rigorous safety and tasting standards (*20*).

However, whether consumers on a gluten-free diet actually believe the food to be sufficiently safe is another question. This mistrust on the part of consumers is based on the fact that gluten (>20 mg/kg) is also found in foods that are labelled as gluten-free (*9*, *11*). In addition, naturally gluten-free foods may bear the label ‘may contain traces of gluten’ if the manufacturers think cross-contact is possible, which may leave consumers even more confused and uncertain about whether they can safely consume the food. This doubt, perceived by individuals on a gluten-free diet, determines whether they will choose to consume the food. Despite increasing market availability, gluten-free products remain a concern due to limited consumer understanding of labelling and the risk of cross-contamination and mislabelling (*21*).

Concerns about possible gluten contamination in naturally gluten-free foods, such as rice, can undermine consumer confidence. It is therefore important to investigate how such concerns shape individual risk perceptions and subsequently influence food choice and adherence to dietary restrictions. To fill this knowledge gap, a two-pronged approach was taken in the present study. First, an online questionnaire was used to assess the perceived risk of gluten contamination in rice among individuals following a gluten-free diet, and second, laboratory analyses were used to determine the actual presence of gluten in rice samples available on the Croatian market. The aim of this study is to compare the perceived risk among consumers with the actual risk of gluten contamination.

## MATERIALS AND METHODS

### Rice sampling and ELISA assay for gluten contamination

Forty-one rice samples from six manufacturers were randomly sampled from 78 rice products available on the Croatian market at the time of this study, including white rice, integral rice and parboiled rice. All samples were obtained from local supermarkets in 2020 and were imports because rice is not cultivated commercially in the country (Table S1).

Each of the 41 rice samples was ground to a fine powder using a grinder, sieved through a 0.5 mm mesh sieve and stored in a plastic tube at room temperature. After each sample had been processed, the grinder, sieve, metal spoon and metal scissors were thoroughly washed, disinfected with 70 % alcohol and dried before the next sample.

Each rice product was analysed in duplicate for the presence of gluten using the RIDASCREEN® Gliadin sandwich R5 enzyme-linked immunosorbent assay - ELISA (R-Biopharm, Darmstadt, Germany) according to the manufacturer's instructions as described below. Powdered samples (0.25 g) were weighed out using an analytical balance and placed in test tubes for analysis. A volume of 2.5 mL of RIDASCREEN® cocktail solution was added to the test tube with rice, the solution was gently mixed and the tube was incubated in a water bath at 50 °C for 40 min. The samples were allowed to sit at room temperature for 10 min, then 7.5 mL of 80 % ethanol were added and the mixture was left on a shaker for 60 min at room temperature. The samples were centrifuged (Rotofix 32 A; Hettich, Tuttlingen, Germany) for 10 min at 2500×*g* at room temperature, and an aliquot (80 µL) of the supernatant was diluted with RIDASCREEN® buffer for subsequent assay. As a positive control, gluten-containing FAPAS® Quality Control Cake Mix (Fera Science, York, UK) was used.

Concentrated solutions of the buffer, conjugate and wash buffer from the above-mentioned kit were diluted according to the manufacturer's instructions. Assay plates coated with R5 anti-gliadin antibodies were filled with 100 µL of manufacturer-supplied standard (0 to 80 ng/mL gliadins) or sample solution in duplicate, and plates were incubated for 30 min at room temperature. Wells were washed three times with 250 µL of diluted wash buffer, then 100 µL of enzyme conjugate were added to each well and the plates were incubated for 30 min at room temperature. Each well was washed three times with 250 µL of wash buffer, then 50 µL of substrate and 50 µL of chromogen were added to each well, and plates were incubated for 30 min at room temperature in the dark. Finally, a stop solution was added and absorbance was measured at 450 nm using a microplate spectrophotometer (absorbance microplate reader ELx800; BioTek Instruments, Inc., Winooski, VT, USA). Results were generated using RIDASOFT® Win software (R-Biopharm). Gluten results are expressed as mass fraction in milligrams of gluten per kilogram of sample (mg/kg), in accordance with the ELISA method used (RIDASCREEN® Gliadin). Values were calculated based on gliadin content and multiplied by a factor of 2, as recommended, to obtain the total gluten content. The limits of detection (LoD) and limits of quantitation (LoQ) were defined according to the manufacturer's instructions for the RIDASCREEN® Gliadin ELISA kit (R-Biopharm). The LoD was 0.5 mg/kg of gliadin, which corresponds to 1 mg/kg of gluten. The LoQ was 2.5 mg/kg of gliadin, equivalent to 5 mg/kg of gluten.

According to Codex Alimentarius (*19*) and EU Regulation No 828/2014 (*18*), the threshold for a product to be considered gluten-free is <20 mg/kg of gluten. This limit was used as a reference point for interpreting the analytical results.

### Survey of consumers who follow a gluten-free diet

For this cross-sectional study, 66 individuals, of which 88 % were female, adhering to a gluten-free diet were recruited through the local coeliac society, representing approx. 67 % of its membership. Data were collected between April and May 2020. Of the respondents, 46 adults completed the online questionnaire independently, while caregivers completed the questionnaire on behalf of 20 minors who follow a gluten-free diet. The questionnaire was completed anonymously *via* an online platform to ensure voluntary participation. Before accessing the survey, all participants gave their informed consent electronically; those who refused consent were kindly asked to leave the survey link. The study was approved by the Ethical Committee of the Faculty of Food Technology and Biotechnology, University of Zagreb (Zagreb, Croatia) under the number 251-69-12-25-16.

### Questionnaire

The questionnaire comprised a total of 29 questions, of which 9 were open- and 20 closed-ended questions. The questionnaire assessed knowledge, behaviour and beliefs about a gluten-free diet, as well as the perception of the risk of gluten contamination in rice. Part of the survey was the Gluten-Free Eating Assessment Tool (*22*), which was designed to assess self-reported adherence to a gluten-free diet, perceived risk of gluten exposure, and dietary knowledge and behaviour to identify areas where individuals may need additional support or education and where they may experience difficulties related to a gluten-free diet (*23*). In addition to the standard questionnaire questions, we added questions about beliefs and consumption of gluten-free food, including questions assessing the perceived risk of gluten contamination in rice and other naturally gluten-free food. Two questions assessed the level of trust in gluten-free labelling using a 5-point Likert scale (1=minimum trust to 5=maximum trust). Participants were asked to indicate the extent to which they trust manufacturers that use the crossed grain symbol on their products and those who use the gluten-free label on the packaging.

### Statistical analysis

Data were reported as *N* (%), mean±standard deviation (S.D.) or median and interquartile range (IQR), as appropriate. Chi-square test was used to assess the difference in trust between the crossed grain symbol and the gluten-free label, with p<0.05 interpreted as indicating a significant difference. Data were analysed statistically using SPSS v. 23.0 (Chicago, IL, USA).

## RESULTS AND DISCUSSION

### Analysis of gluten contamination in rice on the Croatian market

**Fig. 1:**
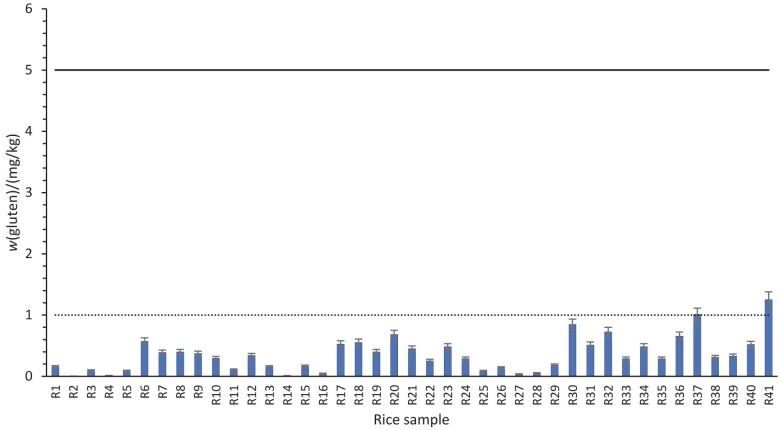
Testing of rice on the Croatian market for the presence of contaminating gluten. Results are shown for duplicate samples. The horizontal dotted line indicates the limit of detection (LOD), while the horizontal solid line indicates the limit of quantification (LOQ). Samples are described in detail in Table S1

Gluten was not detected in any of the 41 samples of white, brown or parboiled rice, based on a validated analytical method with a limit of quantification of 5 mg/kg (Fig. 1). The rice used in samples was cultivated in Italy, Pakistan, India, France and elsewhere in the European Union. For this reason, the results are not only relevant to the Croatian market where the study was conducted, but may also reflect a broader issue relevant to international food safety and trade. The results suggest that the rice available on the market is gluten-free and does not pose risks to individuals following a gluten-free diet. These results are in contrast to some studies that have found a low frequency and level of contamination in rice on the Italian and Swedish markets (*9*, *24*). Strørsud *et al*. (*24*) found that out of 7 rice samples, 5 contained less than 20 mg/kg gluten, and two samples contained between 20 and 200 mg/kg. Verma *et al.* (*9*) found gluten contamination >20 mg/kg only in one of 24 rice samples. Even the occasional contamination in these studies highlights that the risk of contamination is real. Indeed, a study in the USA did not detect gluten contamination in rice, but it did in other naturally gluten-free grains such as millet (*8*), reflecting the potential for cross-contamination during cultivation, processing, transport and storage. Similarly, a study conducted in Canada showed that naturally gluten-free flour is not exempt from this risk. It reported that 9.5 % of samples had gluten mass fractions above 20 mg/kg, including rice flour, with gluten mass fractions ranging from 6 to 1485 mg/kg in brown rice flour (*25*). In another study, gluten contamination was even found in prepared rice-based dishes such as risotto. Although the gluten content in the samples was below 20 mg/kg, the estimated intake per serving was 3.45 mg gluten (*26*). These observations highlight the importance of robust quality controls throughout the food supply chain.

### Survey of Croatians on a gluten-free diet and their perception of rice as gluten-free

The sample of our survey, which was predominantly female (58 of 66 respondents), was (38±8) years old and included 20 minors whose caregivers completed the questionnaire on their behalf (Table 1). Respondents reported having followed a gluten-free diet for a median of 3 years (interquartile range, 1.5–5.0), indicating relatively long-term commitment and experience. Most respondents reported following a gluten-free diet because of coeliac disease (*N*=49), while a smaller number cited various other reasons. This is in line with general trends, which show that the market for gluten-free foods is growing (*4*), not only because of diagnosed cases of coeliac disease, but also because more and more people believe they have non-coeliac gluten sensitivity (*6*, *7*) and choose gluten-free products for the perceived health benefits (*5*).

**Table 1:** Questionnaire items assessing characteristics and dietary practices of individuals on a gluten-free diet in our cross-sectional survey (*N*=66)

Question	Response	*N*/%
What are the reason(s) that you follow a gluten-free diet?*	Autism	0
	Dermatitis herpetiformis	5
	Coeliac disease	59
	Allergy/sensitivity	16
	Inflammatory bowel syndrome	8
	Healthier lifestyle	7
	Body weight control	2
	Other	2
Have you had a duodenal biopsy to look for celiac disease?	Yes	90
	No	10
Please describe your current diet	No restrictions	1
	Strictly gluten-free	80
	Usually gluten-free with rare intentional gluten consumption	5
	Usually gluten-free with rare unintentional gluten consumption	9
	Trying to be gluten-free but not always sure	5
		
Do you avoid or restrict any food(s) other than gluten?	Yes	65
	No	35
What is your main source of information about a gluten-free diet?*	Family physician	3
	Internet	28
	Dietician/nutritionist	5
	Cookbook	4
	Coeliac support group	19
	Print media	2
	Other patients with coeliac disease	24
	Gastroenterologist	12
	Medical book	3

*Respondents could select multiple answers

**Fig. 2:**
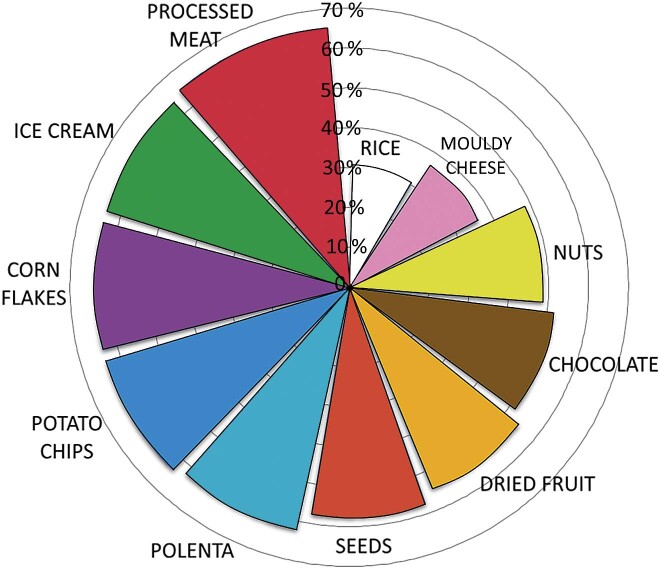
Percentage of individuals avoiding food due to fear of gluten contamination among individuals on a gluten-free diet (*N*=66)

Of the 66 respondents, most (68 %) reported that they were uncertain whether the rice that they consumed was contaminated with gluten and only 12 felt certain it was not. The high level of uncertainty among consumers about gluten contamination in rice, although we demonstrated a low risk, could affect dietary choices. Many people trying to adhere to a gluten-free diet follow the ’when in doubt, avoid it’ rule, so if they see a ’gluten-free’ label on a food, which is not usually found on the packaging of naturally gluten-free foods such as rice, they may prefer to avoid it altogether. Surprisingly, when participants were asked to indicate which packaged foods — that are not specifically labelled as gluten-free or come from trusted manufacturers — they avoid for fear of gluten contamination, rice was the least avoided of the listed food (Fig. 2). This is particularly notable given that the majority of respondents consider rice to be a risk for gluten contamination. This discrepancy highlights the disparity between perceived risk and actual avoidance behaviour and suggests that additional factors may influence dietary choices in this context, including knowledge and awareness of gluten sources (*22*), social influences from family and peers (*27*), economic constraints given the higher cost of gluten-free products (*28*-*30*), all of which may lead to either stricter avoidance or a more relaxed attitude towards a gluten-free diet.

Concerningly, our survey also shows that many naturally gluten-free foods such as processed meat, ice cream, corn flakes, potato chips, polenta, seeds, dried fruit, chocolate, nuts and mouldy cheese are avoided (Fig. 2). These products often fall into a ’grey area’ where the presence of gluten is possible, leading consumers to avoid them altogether. The fact that healthy foods such as seeds, nuts and dried fruit are avoided is worrying, as a gluten-free diet (GFD) is primarily about ensuring that the food is not contaminated with gluten, while nutritional quality does not seem to be a priority. This avoidance may be a major reason why the gluten-free diet is unbalanced and why up to 38 % of people on a gluten-free diet are deficient in macro- or micronutrients (*30*-*34*).

The perception gap regarding gluten contamination is critical because it shows that objective scientific evidence alone is not sufficient to influence consumer beliefs, especially in sensitive areas such as dietary restrictions related to health issues. Research on risk perception supports this idea and shows that subjective factors such as social influence and trust in information sources often carry more weight than objective data (*35*). In today's digital age, the internet is the main source of information for many, but it is a platform where the accuracy and reliability of content can be questionable. Notably, the internet was the primary source of information for most respondents (82 %), followed by peer groups (Table 2). Indeed, a significant number of people diagnosed with coeliac disease shared their experiences and difficulties on TikTok, demonstrating how social media serves as a platform for patients to connect and seek support (*36*). Studies show that while platforms are effective in disseminating information, they also spread misleading content and questionable accuracy, which has a significant potential to mislead people following a gluten-free diet (*37*-*39*). This emphasises the strong influence of informal networks, including social media, where misinformation or anecdotal evidence can spread rapidly and shape collective beliefs.

**Table 2:** Cross-sectional survey on beliefs about gluten contamination among the individuals on a gluten-free diet (*N*=66)

Question	Response	*N*/%
Do you consume naturally gluten-free grains?	Yes	91
	No	9
		
Do you consume products labelled with ‘May contain gluten’?	Yes	41
	No	59
Are you satisfied with gluten labelling on food products?	Yes	24
	No	76
Is raw rice on the market contaminated with gluten?	Yes	14
	No	18
	Not sure	68
		Mean value±S.D.
How much do you trust the ’crossed grain’ symbol indicating that a food is gluten-free?	Scale from 1 (minimal trust) to 5 (maximal trust)	(4.2±0.9)^#^
How much do you trust the label ’Gluten-free’?	Scale from 1 (minimal trust) to 5 (maximal trust)	(3.6±1.0)^#^

^#^These two values differ significantly from each other according to the χ^2^ test (p<0.05)

Respondents expressed significantly higher trust in a symbol indicating gluten-free food than in the word-based ’gluten-free’ label (p<0.05), but only 59 % of respondents said they did not consume foods labelled with ’may contain gluten’ and 76 % of respondents said they were generally dissatisfied with gluten labelling on food products (Table 2). Clear and consistent communication about both the inherently gluten-free nature of rice and other gluten-free grains and the risk of cross-contamination, which requires careful processing and handling, is crucial to reduce consumer anxiety and promote informed decision-making. Part of this communication involves easily understandable labelling of foods, but most respondents expressed overall dissatisfaction with the labelling of gluten-free products, despite European regulations on allergen labelling (*40*) and gluten labelling (*18*). According to these regulations, gluten-free foods such as rice can, of course, be labelled as gluten-free, but such labelling imposes additional costs and responsibility for the manufacturer to check whether the food is gluten-free and to prevent cross-contamination during the production process. Our survey showed that respondents trust the crossed grain symbol much more than the ’gluten-free’ label (p<0.05), which could be due to the fact that the crossed grain symbol certified by AOECS involves strict certification and compliance checks (*20*), whereas the general ’gluten-free’ label indicates compliance with legal limits without necessarily implying third-party verification. Whether this stronger trust reflects an actual lower risk of gluten contamination should be investigated in future work.

### Strengths and limitations

The analysis of rice on the Croatian market included samples from different producers and a wide range of processing methods. Nevertheless, some limitations should be considered when interpreting the results. The survey had a relatively small sample size (*N*=66) and was based on self-reporting, which may be subject to recall bias. However, as the questionnaire was conducted online and anonymously, this likely reduced social desirability bias and encouraged more honest responses from participants. In addition, the sample was predominantly female, which, although consistent with the previously reported ratio of women to men in adherence to the gluten-free diet (*41*), may limit the generalisability of the results to the wider population. Regarding the method of analysis, gluten content was measured with a single ELISA kit. The various commercial ELISA kits differ in their gluten detection due to differences in antibodies and extraction methods, leading to inconsistent results across platforms. Problems such as matrix effects and extraction inefficiencies, which often occur with complex or processed foods, are less relevant with raw rice samples. However, the lack of standardised calibration and the varying sensitivity of the kits can affect the accuracy of gluten quantification (*42*).

## CONCLUSIONS

The results of this study show that the rice samples tested contain no detectable amounts of gluten. This indicates a low or negligible risk of gluten contamination and reassures people who follow a gluten-free diet. However, the majority of respondents who follow a gluten-free diet are unsure about the risk of gluten contamination in rice, which emphasises the need for solutions that facilitate the selection of foods that are safe for consumption. Clearly, future research should investigate not only how the risk of gluten contamination in rice and other naturally gluten-free grains can be minimised to reduce consumer anxiety, but also how perceived risk influences food choices and ultimately affects the overall quality of the diet of individuals who follow a gluten-free diet.

## Appendix

**Table S1:** Rice samples used in this study

Manufacturer	Sample ID	Product name	Country of manufacturer	Country of rice origin
Manufacturer 1	R1	White, medium grain rice (Arborio)	Slovenia	Italy
Manufacturer 1	R2	White, round grain rice	Slovenia	Italy
Manufacturer 1	R3	Parboiled, white long grain rice	Slovenia	Italy
Manufacturer 1	R4	Brown, parboiled rice	Slovenia	Italy
Manufacturer 1	R5	White, round grain rice, organic	Slovenia	Italy
Manufacturer 1	R6	White, medium grain rice	Slovenia	Italy
Manufacturer 1	R7	Basmati, white long grain rice	Slovenia	Pakistan
Manufacturer 1	R8	White, round grain rice	Slovenia	Italy
Manufacturer 1	R9	White, round grain rice	Slovenia	Italy
Manufacturer 1	R10	White, long grain rice	Slovenia	Italy
Manufacturer 1	R11	Mix of brown, red and black whole grain rice	Slovenia	Italy
Manufacturer 2	R12	Parboiled, white, long grain (Oro)	Italy	Italy
Manufacturer 2	R13	Parboiled, white long grain rice	Italy	Italy
Manufacturer 2	R14	White, long grain rice	Italy	Italy
Manufacturer 2	R15	Basmati rice	Italy	Italy
Manufacturer 2	R16	White, long grain rice	Italy	Italy
Manufacturer 2	R17	White, long grain rice (Sant’ Andrea)	Italy	Italy
Manufacturer 2	R18	White, long grain rice (Arborio)	Italy	Italy
Manufacturer 2	R19	White, long grain rice (Roma)	Italy	Italy
Manufacturer 2	R20	White, long grain rice	Italy	Italy
Manufacturer 3	R21	Brown rice, long grain rice	Italy	Italy
Manufacturer 3	R22	Parboiled, long grain rice	Italy	Italy
Manufacturer 3	R23	Basmati, white, long grain rice	Italy	Italy
Manufacturer 3	R24	White, long grain rice	Italy	Italy
Manufacturer 4	R25	White, long grain rice (Arborio)	Italy	Italy
Manufacturer 4	R26	White, round grain rice	Italy	Italy
Manufacturer 4	R27	White, long grain rice (Roma)	Italy	Italy
Manufacturer 4	R28	White, short round grain rice (Originario)	Italy	Italy
Manufacturer 4	R29	White, long grain rice	Italy	Italy
Manufacturer 4	R30	Brown rice	Italy	Italy
Manufacturer 4	R31	White, long grain rice (Carnaroli)	Italy	Italy
Manufacturer 4	R32	Parboiled, long grain rice	Italy	Italy
Manufacturer 4	R33	Red rice	Italy	France
Manufacturer 4	R34	Brown, long grain rice	Italy	Italy
Manufacturer 4	R35	White, basmati, long grain rice	Italy	India
Manufacturer 5	R36	White, long grain rice (Arborio)	Croatia	EU
Manufacturer 5	R37	Parboiled, long grain rice	Croatia	EU
Manufacturer 5	R38	White, long grain rice	Italy	Italy
Manufacturer 5	R39	Brown, long grain rice	Italy	India
Manufacturer 5	R40	White, long grain rice (Roma)	Italy	Italy
Manufacturer 6	R41	White, long grain rice	Italy	Italy

## References

[1] Fukagawa NK Ziska LH. Rice: Importance for global nutrition. J Nutr Sci Vitaminol (Tokyo). 2019;65:S2–3. 10.3177/jnsv.65.S2 31619630

[2] Cenni S Sesenna V Boiardi G Casertano M Russo G Reginelli A The role of gluten in gastrointestinal disorders: A review. Nutrients. 2023;15(7):1615. 10.3390/nu15071615 37049456 PMC10096482

[3] Kurppa K Mulder CJ Stordal K Kaukinen K. Celiac disease affects 1% of global population: Who will manage all these patients? Gastroenterology. 2024;167(1):148–58. 10.1053/j.gastro.2023.12.026 38290622

[4] Gluten-free products market size, share & trends analysis report by product (bakery products, condiments, seasonings, spreads), by distribution channel (convenience stores, supermarkets & hypermarket, specialty stores, online), by region, and segment forecasts, 2025 – 2030. San Francisco, CA, USA: Grand View Research; 2025. Available from: https://www.grandviewresearch.com/industry-analysis/gluten-free-products-market.

[5] Prada M Godinho C Rodrigues DL Lopes C Garrido MV. The impact of a gluten-free claim on the perceived healthfulness, calories, level of processing and expected taste of food products. Food Qual Prefer. 2019;73:284–7. 10.1016/j.foodqual.2018.10.013

[6] Volta U Caio G Tovoli F De Giorgio R. Non-celiac gluten sensitivity: Questions still to be answered despite increasing awareness. Cell Mol Immunol. 2013;10:383–92. 10.1038/cmi.2013.28 23934026 PMC4003198

[7] Volta U De Giorgio R Caio G Uhde M Manfredini R Alaedini A. Non-celiac wheat sensitivity: An immune-mediated condition with systemic manifestations. Gastroenterol Clin North Am. 2019;48(1):165–82. 10.1016/j.gtc.2018.09.012 30711208 PMC6364564

[8] Thompson T Lee AR Grace T. Gluten contamination of grains, seeds, and flours in the United States: A pilot study. J Am Diet Assoc. 2010;110(6):937–40. 10.1016/j.jada.2010.03.014 20497786

[9] Verma AK Gatti S Galeazzi T Monachesi C Padella L Del Baldo G Gluten contamination in naturally or labeled gluten-free products marketed in Italy. Nutrients. 2017;9(2):115. 10.3390/nu9020115 28178205 PMC5331546

[10] Gélinas P McKinnon CM Mena MC Méndez E. Gluten contamination of cereal foods in Canada. Int J Food Sci Technol. 2008;43(7):1245–52. 10.1111/j.1365-2621.2007.01599.x

[11] Lee HJ Anderson Z Ryu D. Gluten contamination in foods labeled as “gluten free” in the United States. J Food Prot. 2014;77(10):1830–4. 10.4315/0362-028X.JFP-14-149 25285507

[12] Guennouni M Admou B El Khoudri N Bourrhouat A Zogaam LG Elmoumou L Gluten contamination in labelled gluten-free, naturally gluten-free and meals in food services in low-, middle- and high-income countries: A systematic review and meta-analysis. Br J Nutr. 2022;127(10):1528–42. 10.1017/S0007114521002488 34753529

[13] Farage P de Medeiros Nóbrega YK Pratesi R Gandolfi L Assunção P Zandonadi RP. Gluten contamination in gluten-free bakery products: A risk for coeliac disease patients. Public Health Nutr. 2017;20(3):413–6. 10.1017/S1368980016002433 27630026 PMC10261395

[14] Yu JM Lee JH Park JD Choi YS Sung JM Jang HW. Analyzing gluten content in various food products using different types of ELISA test kits. Foods. 2021;10(1):108. 10.3390/foods10010108 33419186 PMC7825509

[15] Türköz Bakırcı G Öncü Glaue Ş Akcan T. Determination of gluten contamination in foods available on the Turkish market via Enzyme-Linked Immunosorbent Assay (ELISA). Appl Sci (Basel). 2023;13(10):6143. 10.3390/app13106143

[16] Bustamante MÁ Fernández-Gil MP Churruca I Miranda J Lasa A Navarro V, Simón E Evolution of gluten content in cereal-based gluten-free products: An overview from 1998 to 2016. Nutrients. 2017;9(1):21. 10.3390/nu9010021 28054938 PMC5295065

[17] Wieser H Segura V Ruiz-Carnicer Á Sousa C Comino I. Food safety and cross-contamination of gluten-free products: A narrative review. Nutrients. 2021;13(7):2244. 10.3390/nu13072244 34210037 PMC8308338

[18] Commission implementing regulation (EU) No 828/2014 of 30 July 2014 on the requirements for the provision of information to consumers on the absence or reduced presence of gluten in food. OJ L. 2014;228:5-8. Available from: https://eur-lex.europa.eu/legal-content/HR/ALL/?uri=celex:32014R0828.

[19] CXS 118-1979. Standard for foods for special dietary use for persons intolerant to gluten. Codex Alimentarius. Geneva, Switzerland: Food and Agriculture Organization of the United Nations and World Health Organization (FAO/WHO); 2015. Available from: https://www.fao.org/fao-who-codexalimentarius/codex-texts/list-standards/en/.

[20] Gluten free certification. Brussels, Belgium: Association of Europen Coeliac Societes (AOECS); 2012. Available from: https://www.aoecs.org/working-with-food/gluten-free-certification/.

[21] Hassan HF Mourad L Khatib N Assi R Akil S El Khatib S Perceptions towards gluten free products among consumers: A narrative review. Appl Food Res. 2024;4(2):100441. 10.1016/j.afres.2024.100441

[22] Silvester JA Weiten D Graff LA Walker JR Duerksen DR. Is it gluten-free? Relationship between self-reported gluten-free diet adherence and knowledge of gluten content of foods. Nutrition. 2016;32(7-8):777–83. 10.1016/j.nut.2016.01.021 27131408 PMC5457910

[23] Farage P Zandonadi R. The gluten-free diet: Difficulties celiac disease patients have to face daily. Austin J Nutr Food Sci. 2014;2(5):1027.

[24] Størsrud S Malmheden Yman I Lenner RA. Gluten contamination in oat products and products naturally free from gluten. Eur Food Res Technol. 2003;217(6):481–5. 10.1007/s00217-003-0786-0

[25] Koerner TB Cleroux C Poirier C Cantin I La Vieille S Hayward S Gluten contamination of naturally gluten-free flours and starches used by Canadians with celiac disease. Food Addit Contam Part A Chem Anal Control Expo Risk Assess. 2013;30(12):2017–21. 10.1080/19440049.2013.840744 24124879

[26] Vukman D Viličnik P Vahčić N Lasić D Niseteo T Panjkota Krbavčić I Design and evaluation of an HACCP gluten-free protocol in a children’s hospital. Food Control. 2021;120:107527. 10.1016/j.foodcont.2020.107527

[27] White LE Bannerman E Gillett PM. Coeliac disease and the gluten-free diet: A review of the burdens; factors associated with adherence and impact on health-related quality of life, with specific focus on adolescence. J Hum Nutr Diet. 2016;29(5):593–606. 10.1111/jhn.12375 27214084

[28] Missbach B Schwingshackl L Billmann A Mystek A Hickelsberger M Bauer G, König J Gluten-free food database: The nutritional quality and cost of packaged gluten-free foods. PeerJ. 2015;3:e1337. 10.7717/peerj.1337 26528408 PMC4627916

[29] Fry L Madden AM Fallaize R. An investigation into the nutritional composition and cost of gluten-free versus regular food products in the UK. J Hum Nutr Diet. 2018;31(1):108–20. 10.1111/jhn.12502 28851025

[30] Mehtab W Agarwal S Agarwal H Ahmed A Agarwal A Prasad S Gluten-free foods are expensive and nutritionally imbalanced than their gluten-containing counterparts. Indian J Gastroenterol. 2024;43(3):668–78. 10.1007/s12664-024-01519-z 38753225

[31] Jivraj A Hutchinson JM Ching E Marwaha A Verdu EF Armstrong D Micronutrient deficiencies are frequent in adult patients with and without celiac disease on a gluten-free diet, regardless of duration and adherence to the diet. Nutrition. 2022;103–104:111809. 10.1016/j.nut.2022.111809 36096056

[32] Theethira TG Dennis M Leffler DA. Nutritional consequences of celiac disease and the gluten-free diet. Expert Rev Gastroenterol Hepatol. 2014;8(2):123–9. 10.1586/17474124.2014.876360 24417260

[33] Vici G Belli L Biondi M Polzonetti V. Gluten free diet and nutrient deficiencies: A review. Clin Nutr. 2016;35(6):1236–41. 10.1016/j.clnu.2016.05.002 27211234

[34] Bituh M Žižić V Panjkota Krbavčić I Zadro Z Barić IC. Gluten-free products are insufficient source of folate and vitamin B12 for coeliac patients. Food Technol Biotechnol. 2011;49(4):511–6.

[35] Santeramo FG Lamonaca E. Objective risk and subjective risk: The role of information in food supply chains. Food Res Int. 2021;139:109962. 10.1016/j.foodres.2020.109962 33509512

[36] Jaime C Samuel L Fera J Basch CH. Discussing health while seeking community: A descriptive study of celiac disease on TikTok. Nutr Health. 2023;29(1):37–41. 10.1177/02601060221127505 36148909

[37] Al Sarkhy A. Social media usage pattern and its influencing factors among celiac patients and their families. Saudi J Gastroenterol. 2020;26(2):99–104. 10.4103/sjg.SJG_495_19 32031161 PMC7279077

[38] Park K Harris M Khavari N Khosla C. Rationale for using social media to collect patient-reported outcomes in patients with celiac disease. J Gastrointest Dig Syst. 2014;4(1):166. 10.4172/2161-069X.1000166 25392743 PMC4226063

[39] Verma AK Quattrini S Serin Y Monachesi C Catassi GN Gatti S Unauthentic information about celiac disease on social networking pages: Is it a matter of concern in celiac disease management? Dig Dis Sci. 2024;69(10):3650–60. 10.1007/s10620-024-08486-7 38816597

[40] Regulation (EU) No 1169/2011 of the European Parliament and of the Council of 25 October 2011 on the provision of food information to consumers, amending Regulations (EC) No 1924/2006 and (EC) No 1925/2006 of the European Parliament and of the Council, and repealing Commission Directive 87/250/EEC, Council Directive 90/496/EEC, Commission Directive 1999/10/EC, Directive 2000/13/EC of the European Parliament and of the Council, Commission Directives 2002/67/EC and 2008/5/EC and Commission Regulation (EC) No 608/2004. OJ L. 2011;304:18-63. Available from: https://eur-lex.europa.eu/legal-content/HR/ALL/?uri=celex:32011R1169.

[41] Jansson-Knodell CL Hujoel IA West CP Taneja V Prokop LJ Rubio-Tapia A Sex difference in celiac disease in undiagnosed populations: A systematic review and meta-analysis. Clin Gastroenterol Hepatol. 2019;17(10):1954–1968.e13. 10.1016/j.cgh.2018.11.013 30448593

[42] Amnuaycheewa P Niemann L Goodman RE Baumert JL Taylor SL. Challenges in gluten analysis: A comparison of four commercial sandwich ELISA kits. Foods. 2022;11(5):706. 10.3390/foods11050706 35267339 PMC8909647

